# EdU-Based Step-by-Step Method for the Detection of Sister Chromatid Exchanges for Application in Plant Genotoxicity Assessment

**DOI:** 10.3389/fpls.2020.01146

**Published:** 2020-07-24

**Authors:** Jolanta Kwasniewska, Adrianna Bara

**Affiliations:** Plant Cytogenetics and Molecular Biology Group, University of Silesia in Katowice, Katowice, Poland

**Keywords:** 5-ethynyl-2′-deoxyuridine, sister chromatid exchange, genotoxicity, maleic hydrazide, gamma-ray

## Abstract

This study is an example of using 5-ethynyl-2′-deoxyuridine (EdU) for detecting sister chromatid exchanges (SCEs) at chromosomal level. Here we report a detailed protocol for differential labeling sister chromatids in barley (*Hordeum vulgare*, 2n = 14) cells that is based on the incorporation and simple detection of EdU. The perfect distinguishing of sister chromatids enabled an analysis of the effects of two model agents—maleic acid hydrazide (MH) and gamma rays—on the formation of SCEs. Using this method, we demonstrated the high sensitivity of barley cells to maleic hydrazide, which is expressed as an increased level of SCEs. A gamma ray induced only slightly more SCEs than in the control cells. The possible mechanisms of MH and gamma ray action in respect to distinguishing chromatids using EdU are discussed. Recommendation for SCEs visualization using EdU as an easy and quick method that can be successfully adapted to other plant species and potentially for human genotoxicity studies is presented.

## Introduction

The consequences of the influence of environmental factors are detected using cytogenetic and molecular biological markers. Among these, a sister chromatid exchange (SCE) test is considered to be one of the most sensitive cytogenetic methods that is commonly used to estimate the genotoxic effect of different mutagens. SCEs show the interchange mechanism between the sister chromatids of mitotic chromosomes. A number of detection techniques have been used to visualize SCEs in the linear chromosomes. The occurrence of SCEs was first demonstrated in plant cells through the autoradiographic analysis of tritium-labeled chromosomal DNA at a very low resolution due to size of grains and the localization of their spread (Taylor, 1958). Recognizing small SCEs was impossible using this method. It was significantly improved after the method that is based on the incorporation of the halogenated thymidine analog 5-bromodeoxyuridine (BrdU) was developed (Latt, 1973). After BrdU is incorporated, the differential staining of SCEs can subsequently be achieved by using different methods: modified Giemsa staining (Korenberg and Freedlender, 1974), 33258 Hoechst (Perry and Wolff, 1974), acridine orange or 4′-6′-diamidino-2-phenylindole (DAPI) (Lin and Alfi, 1976). These approaches, which are based on BrdU as a label, together with the simplicity and low number of cells that need to be scored have made SCEs the preferred end point in mutagenesis studies.

Unfortunately, the impact of BrdU on the spontaneous level of SCEs has previously been shown (Natarajan et al., 1986) in both control and mutagen-treated cells. It is not clear whether BrdU causes DNA damage or affects the effectiveness of repairs after mutagenic treatment. Among the mutagens, BrdU strongly enhances the frequency of the SCEs that are induced by UV radiation (Wojcik et al., 2003). Moreover, 5-fluorodeoxyuridine (FdU) when added to a BrdU solution in order to inhibit the endogenous synthesis of thymidylic acid in plant cells and to enhance the incorporation of BrdU was proven to increase the frequency of SCEs (Kilhman and Kronborg, 1975; Hongju and Zili, 1992). An alternative, relatively new method for the differential staining of chromatids that is based on biotin-2′-deoxyuridine-5′-triphosphate (dUTP), which is detected using immunological methods, also has consequences such as a higher level of SCEs compared to the level of SCEs that can be obtained using the lowest applicable concentration of BrdU. The reason is that there is some steric hindrance during DNA replication (Bruckmann et al., 1999). There are also some disadvantages such as the necessity of using strong denaturation during the BrdU detection procedure, which influences the chromatin structure and the relatively large signal size of the specific antibodies that are used for the detection of BrdU. For the last several years, BrdU has also been used to reveal replicated chromatin. Recently, the “click” reaction with 5-ethynyl-2′-deoxyuridine (EdU) (Buck et al., 2008; Cavanagh et al., 2011) was successfully introduced to examine the DNA replication pattern in nuclei and chromosomes (Kwasniewska et al., 2016; Kwasniewska et al., 2018) and to differentiate the sister chromatids in animals (Sunada et al., 2019) and plants (Schubert et al., 2016). The sister chromatids were differentiated with EdU application in *Luzula elegans* and rye to study their arrangement in monocentric and holocentric chromosomes (Schubert et al., 2016); however, there are still no examples of using EdU in plants for the study of SCEs induced by chemical and physical agents. EdU is a nucleoside analog of thymidine that is incorporated into the DNA during active DNA synthesis, similar to BrdU (Buck et al., 2008). With this technique, the chromatin structure is well preserved, which makes it a highly resolute method that is universally convenient to use for studies of both monocots and dicots (Kotogány et al., 2010). Although the effects of EdU on cell viability, DNA synthesis, and cell cycle progression have not yet been explored in detail, an application for the assessment of dynamic proliferation in flow cytometry has been found (Diermeier-Daucher et al., 2009).

SCEs have been previously applied to studies of the chromosomes of numerous plant species (Schvartzman, 1987), including *Allium* species (Panda et al., 1996), *Vicia faba* (Huilan and Si, 2007), Tradescantia (Peng and Ma, 1990), and *Hordeum vulgare* (Yi et al., 2005; Andronic et al., 2010). Hundreds of different types of substances have been tested for their mutagenic potential in SCEs. The effectiveness of the different mutagens in the production of SCEs is related to their mechanisms of action and is a consequence of the types of DNA lesions. Among the different mutagen effects, interstrand cross-links are the most probable lesions that lead to the formation of SCEs (Latt, 1981). The wide applications of barley in mutagenesis, which involves the development of new varieties and large chromosomes, make it a convenient model in studies on the effects of mutagens that are observed in chromosomes (Juchimiuk et al., 2007; Juchimiuk-Kwasniewska et al., 2011). Barley is commonly used for root meristem cytogenetic tests and seedling growth assays.

In the present study, a method for analyzing SCEs in barley as a model plant species is presented. The experiments were carried out with two aims: (1) to optimize the SCE method using EdU in barley for genotoxicity studies and (2) to perform a comparative analysis of the frequency of SCEs that are visualized by the incorporation of EdU after mutagenic treatment with maleic acid hydrazide (MH) and gamma ray. Gamma ray and maleic acid hydrazide are routinely used in plant mutagenesis, and many new plant mutant varieties, including barley, have been developed through their application (Hagberg and Persson, 1968; Schulte et al., 2009). Using the approach developed in the present study, its application in studying environmental mutagenesis is considered.

## Materials and Methods

### Plant Material, Treatment, and Growth Conditions

Seeds of the barley (*Hordeum vulgare*, 2n = 14) “Start” variety were used as the plant material.

Maleic acid hydrazide (4 mM MH) and a gamma ray (175 Gy) were used for mutagenic treatment. The mutagen doses used in the study were applied in previous experiments in which their cytogenetic effects in barley were estimated (Juchimiuk et al., 2007; Juchimiuk-Kwasniewska et al., 2011). The irradiation was performed at the International Atomic Energy Agency, Seibersdorf Laboratory, Austria. After irradiation, the seeds were pre-soaked in distilled water for 8 h and germinated in Petri dishes at 21°C in the dark. Before chemical treatment, the seeds of barley were pre-soaked in distilled water for 8 h and then treated with MH for 3 h. After the treatment, the seeds were washed three times in distilled water and then germinated in Petri dishes at 21°C in the dark. The experiment with each mutagen was repeated three times.

### Reagents

- Click-iT EdU Imaging Kits, Alexa Fluor 488 (Invitrogen, Carlsbad, CA, USA, C10337), which contains:
  5-ethynyl-2-deoxyuridine (EdU; component A)
  Alexa Fluor 488 azide (component B)
  Dimethylosulfoxide (DMSO; component C)
  Click-iT reaction buffer (component D)
  Copper sulphate solution (CuSO4; 100 mM, component E)
  Click-iT EdU buffer additive (Component F)
- BSA (Sigma, Cat. No. A7030)
- Cellulase Onozuka (Serva, Heidelberg, Germany, Cat. No. 28302)
- Citric acid (C_6_H_8_O_7_ H_2_O, Sigma-Aldrich, Cat. No.251275)
- Ethanol (POCH, Cat. No. 396480111)
- Glacial acetic acid (Chempur, Cat. No. Cp-A1010)
- Maleic acid hydrazide (4 mM MH; Sigma, Cat. No. D119806, CAS 123-3301)
- Pectinase (Sigma-Aldrich, Cat. No. P4716)
- Potassium chloride (KCl, POCH, Cat. No. 739740114)
- Potassium dihydrogen phosphate (KH_2_PO_4_, POCH, Cat. No. 742020112)
- Sodium chloride (NaCl, POCH, Cat. No. 794121116)
- Tri-Sodium citrate dehydrate (C_6_H_5_O_7_Na_3_·2H_2_O, POCH, Cat. No. 795780112)
- Sodium hydrogen phosphate (Na_2_HPO_4_, POCH, Cat. No. BA9230112)
- Sodium hydroxide (NaOH; Merck, Cat. No.1099130001)
- Triton X-100 (Sigma, Cat. No. T8787)
- Vectashield antifade mounting medium (Vector, Burlingame, CA, USA)

### Equipment

- Aeration pump
- Aluminium foil
- Centrifuge for 50 ml tubes
- Conical flasks 500 ml
- Constant temperature (37°C) incubator
- Cover glass (20 × 20 mm, 24 × 24 mm
- Culture room with controllable temperature and illumination (25 ± 1°C, 16 h/8 h light/dark photoperiod)
- Dry ice
- Eppendorf for 1.5 ml tubes
- Filter paper (20-cm diameter)
- Fine forceps
- Fluorescence microcope with 40× and 100× objectives
- Fridge (4°C) and freezers (−20°C)
- Glass beakers (250 ml)
- Glass bottles (100 ml–1L)
- Glass coplin jar
- Glass Petri dishes (20 cm diameter)
- Glass test tubes (12 ml)
- Graduated cylinders 10–500 ml
- Laboratory microscope with 20× and 40× phase-contrast objectives
- Maceration dish
- Magnetic stirrer
- Micro dissecting needles
- Microscope slides
- Micropipettes and corresponding pipette tips (10 µl–5 ml)
- Microscope slides
- Moist chamber
- pH meter
- Pipetting aid (1–50 ml)
- Plastic foils (24 × 24 mm)
- Stereomicroscope with white light supply unit
- Sterile plastic disposable tubes (20 ml, 50 ml)
- Sterile plastic Petri dishes (9-cm diameter)
- Sterile plastic round bottom centrifuge tubes (50 ml)
- Tissue culture room with controllable temperature and illumination (25 ± 1°C, 16 h/8 h light/dark photoperiod)
- Tweezers
- Vortex mixer

### Reagent Setup

- Acetic acid 45% [100 ml]: 45 ml glacial acetic acid + 55 ml distilled water. Store at room temperature (RT).
- Additive buffer Click-iT EdU [215 μl]: 21.5 μl 10 × additive buffer + 193.5 μl dH_2_O; use fresh.
- BSA 3% [1 ml]: 0.03 g BSA + 1 ml PBS. Prepare immediately before use.
- EdU stock solution 10 mM [2 ml]: 5 mg EdU (Component A) + 2 ml DMSO (component C); mix well, store at −20°C; stock is stable for up to 1 year.
- EdU working solution [500 ml]: 500 μl of 10 mM stock EdU solution + 500 ml distilled water. Use fresh.
- EdU reaction cocktail: for one sample reaction (one chromosome slide), the following components were added: 43 μl of a 1× Click-iT reaction buffer, 2 μl of CuSO4 (Component E, 100 mM), 0.12 μl of Alexa Fluor 488 azide (Component B), and a 5 μl reaction buffer additive (Component F).
- **NOTE**: It is important to add the ingredients in the order listed above; otherwise the reaction will not proceed optimally. The Click-iT reaction buffer should be used within 15 min of preparation.
- Enzyme solution for chromosome preparations [10 ml], 20% pectinase + 2% cellulose: 2 ml pectinase + 0.2 g celullase + up to 10 ml sodium citrate buffer. Store at −20°C; use after heating to 37°C.
- Fixative [200 ml]: methanol:acetic acid (3:1), 150 ml methanol + 50 ml glacial acetic acid. Use fresh.
- MH 4 mM [250 ml]: 0.1121 g MH + 250 ml wody destylowanej. Prepare immediately before use.
- PBS, 1× [250 ml]: 50 mg KCl + 50 mg KH_2_PO_4_ + 2 g NaCl + 720 mg Na_2_HPO_4_ × 12H_2_O + 250 ml dH_2_O; adjust the pH with NaOH to 7.4; store at RT.
- Sodium citrate buffer, stock solution [100 ml]: 40 ml buffer (A) 0.1 M citric acid C_6_H_8_O_7_ × H_2_O (21,01 g/l) + 60 ml buffer (B) 0.1 M sodium citrate C_6_H_5_O_7_Na_3_ × 2H_2_O (29.41 g/l). Store at −20°C.
- Sodium citrate buffer, working solution 0.01 M [100 ml]: 10 ml of sodium citrate buffer stock solution + 90 ml of distilled water. Store at 4°C.
- Triton X-100 0.5%, permeabilized buffer [500 ml]: 500 μl Triton X-100 + 100 ml 1× PBS. Use fresh.

### Step-by-Step Procedure

#### EdU Incorporation (*Timing ~23 h*)

1. Incubate three-day-old barley seedlings in the dark in a 10 mM EdU solution for 11 h (the duration of one barley cell cycle) at 21°C using aeration pump.
2. Wash seedlings in distilled water for 3 min.
3. Transfer seedlings to Petri dishes at 21°C in the dark for the next 11 h.
4. Fix the seedlings in ethanol:glacial acetic acid (3:1) for 2 h at room temperature (RT). Storage at −20°C is possible up to 6 months.

#### Chromosome Preparation From the Roots of the Seedlings (*Timing ~7 h*)

5. Wash the material with a 0.01 mM sodium citrate buffer (pH 4.8) for 30 min.
6. Digest material with 2% cellulase (w/v) and 20% pectinase (v/v) for 2 h at 37°C. The application of this enzyme mixture provides the excellent quality of chromosome preparation: chromosomes are well spread, free of cytoplasm, and clean.

**NOTE:** Prewarm the enzyme solution to 37°C.

7. Wash the material with a sodium citrate buffer for 30 min.
8. Made squash preparations in a drop of 45% acetic acid.
9. Freeze and remove the coverslips.
10. Dry slides for 1 h at RT.

#### EdU Detection (*Timing ~1 h*)

11. Permeabilize the slides with 0.5% Triton X-100 for 20 min.
12. Washed the slides in PBS at RT.
13. Incubate the slide for 30 min at RT in a 50 μl of EdU reaction cocktail.
14. Mount the slides in a Vectashield medium.

#### Analysis (*Timing ~ 5h*)

15. Analyze the slides with a Zeiss Axio Imager.Z.2 wide-field fluorescence microscope equipped with an AxioCam Mrm monochromatic camera, using 495/519 filter.
16. Express the SCEs as the mean number of SCEs per cell, the maximum number of SCEs per chromosome, and the frequency of chromosomes without SCEs that resulted from at least 150 well-spread metaphases for each treatment.
17. Analyze the results by using the statistic test to determine any significant differences (P < 0.05) among the control and treated groups.

## Results

**Figure 1:**
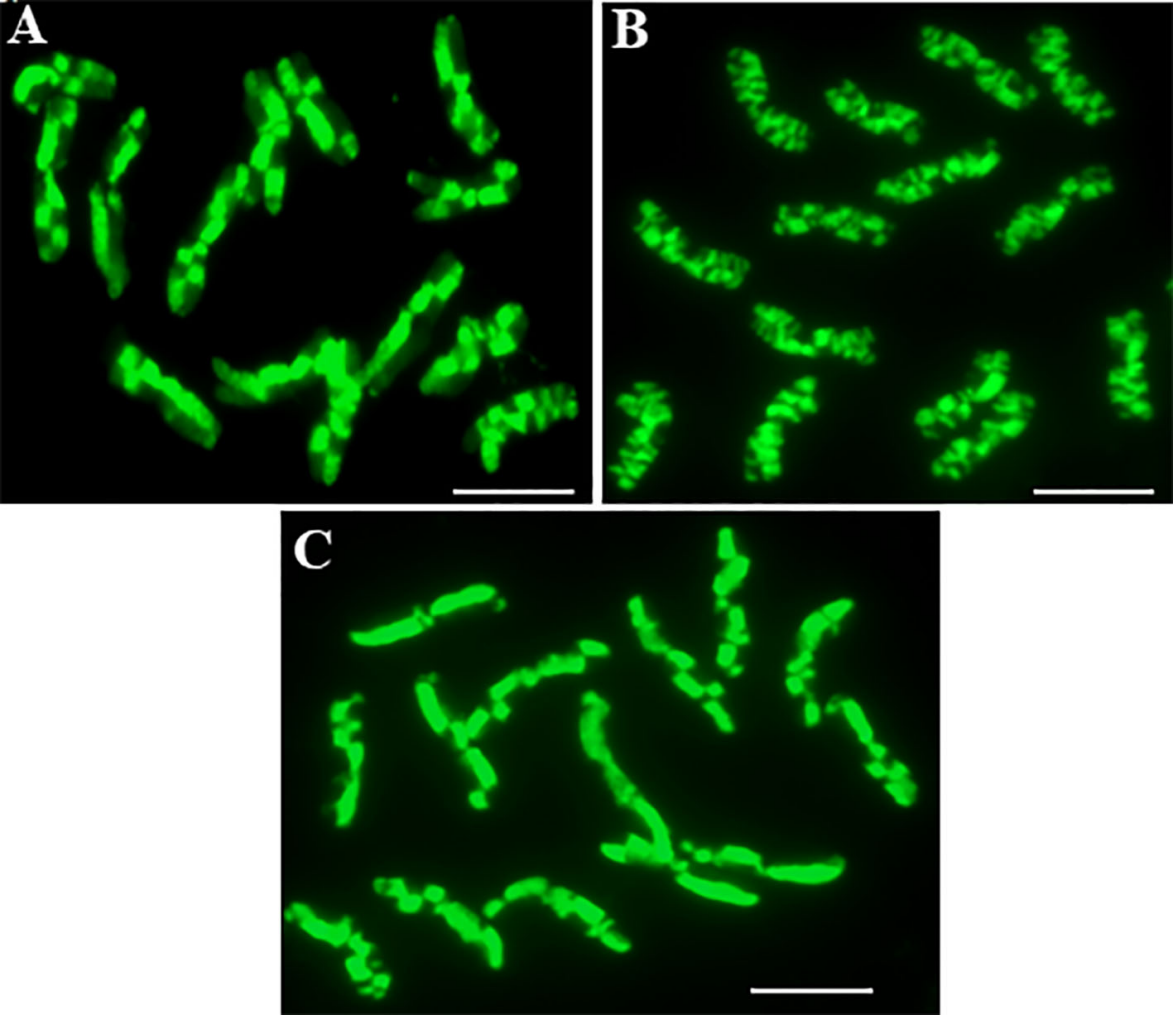
The complete *Hordeum vulgare* (2n = 14) metaphases in root meristematic cells: control **(A)**, after seed treatment with MH **(B)**, after seed irradiation with gamma ray **(C)** showing the SCEs. The EdU-substituted chromatids are characterized by the presence of the green fluorescence of Alexa Fluor 488. Bar represents 5 µm.

**Figure 2:**
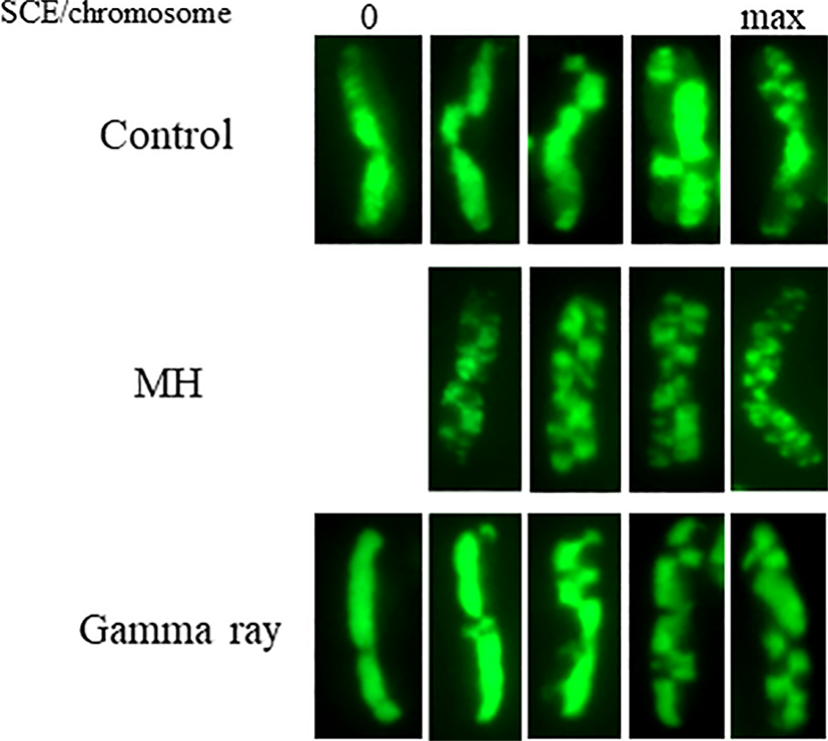
Examples of individual *Hordeum vulgare* (2n = 14) chromosomes with sister chromatid exchanges (SCEs) in control, MH- and gamma-ray-treated root meristem cells. The EdU-substituted chromatids are characterized by the presence of the green fluorescence of Alexa Fluor 488. The presented chromosomes show different number of SCEs: first column—chromosomes without SCEs, last column—chromosomes with the maximum number of SCEs. Bars represent 5 µm.

The application of the method using the incorporation and detection of 5-ethynyl-2-deoxyuridine (EdU) enabled excellent differential staining of the sister chromatid exchanges in the *Hordeum vulgare* “Start” var. (**Figure 1**). Chromatids can be distinguished within one chromosome, and therefore, the SCEs are seen with great clarity and resolution. The unifilarily substituted chromatid (TEdU-TT) is characterized by the green fluorescence of Alexa Fluor 488. The dot-like, small chromatid segments that are being exchanged between the chromatids are also easily distinguished. Therefore, an analysis of the SCEs, which occurred spontaneously (**Figure 1A**), the MH-induced (**Figure 1B**), and gamma ray-induced (**Figure 1C**) in barley root meristems was possible. Due to the similarity of the fourteen barley chromosomes, the level of SCEs was estimated by analyzing the following parameters: the frequency, which is characterized by the number of SCEs per diploid cell, the maximum number of SCEs per chromosome, and the frequency of chromosomes with no SCEs (**Table 1**, **Figure 2**).

**Table 1:** Parameters of sister chromatid exchanges (SCEs) in barley root meristem cells: control and induced by treatment with MH and gamma ray.

**Treatment**	**SCEs frequency (number of SCEs/diploid cell)**	**Maximum number of SCEs/chromosome**	**Frequency of chromosomes without SCEs**
**Control**	38.75 ± 2.5	10	5.25
**4 mM MH** **175 Gy of gamma ray**	59.74 ± 3.6* 42.15 ± 2.8	28 9	0 5.07

*Significant difference from control (P < 0.05).

The results indicate that under control conditions, 38.75 ± 2.5 SCEs occur per diploid cell. The maximum number of SCEs per chromosome was 10, and only 5.25% chromosomes did not show any SCEs in the not-treated cells. Maleic acid hydrazide (MH) significantly increased the level of SCEs. After MH treatment, the frequency of the SCEs increased by 65% and reached 59.74 ± 3.6. The maximum number of SCEs in the MH-treated cells increased almost threefold. At the same time, all of the chromosomes that were observed showed SCEs. In contrast to MH, there was almost no effect of the gamma ray in the dose of 175 Gy that was applied. The frequency of SCEs was only slightly higher than in the control—42.15 ± 2.8. The other SCE parameters were similar to those in the control cells.

## Discussion

It was confirmed that the SCE method using the incorporation of the 5-bromodeoxyuridine (BrdU) in combination with 2′-deoxyuridine (dUrd) and 5-fluoro-2′-deoxyuridine (FdU) increases the sister chromatid exchange yield (Pardo et al., 1987). The application of BrdU can cause a distortion of the chromosome morphology, a lower proportion of metaphase cells per slide, and problems with poor sister chromatid differentiation as a result of an incorrect BrdU concentration (Gerster and Grant, 1989). The elimination of FdU is also not recommended since it has been shown to enhance the uptake of BrdU. Our work presents a step-by-step method for distinguishing sister chromatids to analyze the formation of mutagen-induced SCEs in plant cells for potential use in genotoxicity studies. The results of the present study, which are about the development of a protocol for the differentiation of sister chromatids using EdU after mutagenic treatment, show that chromosome morphology is well preserved. The distribution of SCEs along the length of the chromosomes can be analyzed easily even in mutagen-treated cells. A great clarity and resolution of SCE test with EdU application are especially crucial after mutagenic treatment due to the high level of SCEs and small chromatid segments, which are exchanged. No problems were observed with mitotic activity when EdU was used. The simplicity of the SCE method with EdU should also be emphasized. Differentiation of sister chromatids with EdU was previously applied in *Luzula elegans* and rye (Schubert et al., 2016); however, differences between the protocols for SCE differentiation occur, *e.g.* EdU concentration, time of incubation in EdU solution, and applied post-incubation times. No mutagen-induced SCEs were analyzed previously, which is crucial to test the sensitivity of a new method by using an experimental design to study the response to physical and chemical agents. The SCE test has previously been used in barley using BrdU-substituted chromatids (Schubert et al., 1980; Andronic et al., 2010). Although the results of our studies confirmed the formation of SCEs under control conditions, a direct comparison with previous data is difficult due to differences in the experimental conditions, such as the BrdU concentrations and the time of its incorporation, as well as the different barley varieties and lines that were used for the studies.

Determining the spontaneous level of SCEs still remains a difficult problem. It is still not explained whether SCEs occur spontaneously or whether they are induced by the treatments that are required to differentiate between sister chromatids. Due to the most recent studies using Strand-seq, which is a single-cell DNA template strand sequencing technique, the presence of variable BrdU concentrations has no effect on SCE frequency in either normal or Bloom syndrome cells (Wietmarschen and Lansdorp, 2016). Due to this theory, SCEs reflect the DNA repair events that occur spontaneously. This theory is in contrast to all of the previous hypotheses, which suggest that most of the exchanges that are detected in BrdU-substituted chromosomes, which have not been treated with a mutagen, are BrdU-dependent events. The present study also shows that SCEs occur in control cells even when EdU was used instead of the classical BrdU. Thus, this can either be explained by EdU-dependent damage or as consequence of spontaneously occurring events. The effects of DNA substitution by BrdU on micronucleus induction were shown previously (Weller et al., 1993). We did not observe any changes in the chromosome morphology or the micronuclei, which can indicate its non-mutagenic character. However, further studies are required to confirm our hypothesis.

Although sister chromatid exchanges are induced by chemical agents that produce various types of DNA lesions, not all types of DNA lesions have the same potency to induce SCEs (Latt, 1981). The major DNA lesions that lead to the formation of SCEs are interstrand cross-links. The data that was obtained in this study showed a significant increase in the frequency of SCEs after MH treatment. In contrast to MH, the gamma ray, characterized by the S-independent mode of action, induced only slightly more SCEs than those in the control cells, confirming the previous studies on inducing SCEs by physical agents. SCE formation of chromosomes that are prelabeled with BrdU is strongly dependent on the type of DNA-damaging agent (Stoilov et al., 2002). Upon irradiation, BrdU had a strong effect on SCE induction; BrdU-induced damage is responsible for more than 80% of the SCEs that are formed in UV-irradiated cells that are unifilarily labeled with BrdU (Wojcik et al., 2003). Thus it can be inferred that EdU has no effect on the SCE formation in irradiated cells, as was also shown for BrdU.

Maleic hydrazide (MH) is an S-dependent clastogen, which has been shown to be an inducer of SCEs in plant cells (Cortes et al., 1987). MH induces sister chromatid exchange formation in a similar way to that of alkylating agents (Veselska et al., 1995). It is generally accepted that SCEs are formed during the DNA replication process at the S phase (Gonzalez-Gil and Navarrete, 1986), and therefore, the effect of MH on SCE formation is not surprising. However, in contrast to the gamma ray, no influence of BrdU was shown on SCE formation with MH.

Obviously, more knowledge is needed on SCE formation using the novel protocol with EdU. Optimistically, information about the molecular mechanisms by which SCEs arise in both untreated as well mutagen-treated cells can be obtained. Among them, the formation of BrdU-independent SCEs can be understood.

## Conclusions

This work confirms the usefulness of the EdU method to distinguish sister chromatids. For the first time SCEs using EdU were applied in order to study the mutagen-induced sister chromatid exchanges in plant cells. The great clarity and high resolution with well-preserved chromosomes make the EdU method very convenient for detailed analyses of sister chromatid exchanges, especially if a high number of SCEs occur. SCEs analysis with the incorporation and detection of EdU can be especially suitable for species that have small chromosomes.

## Data Availability Statement

All datasets generated for this study are included in the article/supplementary material.

## Author Contributions

JK conceived and supervised the project. JK and AB are responsible for data curation, formal analysis, and investigation. JK wrote the manuscript. All authors contributed to the article and approved the submitted version.

## Conflict of Interest

The authors declare that the research was conducted in the absence of any commercial or financial relationships that could be construed as a potential conflict of interest.
